# Increased levels of circulating Annexin A5 in Familial Mediterranean fever

**DOI:** 10.1186/1476-9255-7-55

**Published:** 2010-11-23

**Authors:** Anna S Boyajyan, Gohar M Mkrtchyan, Lilit P Hovhannisyan, Tigran J Hovsepyan

**Affiliations:** 1 Institute of Molecular Biology, National Academy of Sciences of the Republic of Armenia 7, Hasratyan St., Yerevan 0014, Armenia

## Abstract

**Background:**

Familial Mediterranean fever is a genetic autoinflammatory disease most commonly affecting the ethnic groups originating from around the Mediterranean Sea. Apoptosis plays an important role in down-regulation of the inflammatory response by reducing the lifespan of activated immunocompetent cells. Thus, increased apoptosis may be associated with pathogenesis of familial Mediterranean fever.

**Methods:**

In the present study we determined the serum levels of apoptotic marker, Annexin A5, in familial Mediterranean fever patients, within an attack and attack-free, in comparison to healthy subjects and assessed the influence of colchicine treatment on this parameter. In addition, in all study subjects serum levels of C-reactive protein and interleukine-1β, and the total leukocyte count were also determined.

**Results:**

Our results demonstrated that pathogenesis of familial Mediterranean fever is characterized by the increased levels of circulating Annexin A5, which is higher in patients within the attack and which associate with the increased levels of C-reactive protein and interleukine-1β and total leukocyte count.

**Conclusions:**

The results obtained indicate elevated rates of apoptosis of subpopulations of leukocytes involved in autoinflammation and recurrent episodes of fever in familial Mediterranean fever. It was also revealed that regular colchicine treatment sufficiently decreases the rate of apoptosis in familial Mediterranean fever patients by affecting the intensity of autoinflammatory reactions.

## Introduction

Familial Mediterranean fever (FMF; MIM 294100) is a genetic autoinflammatory disease most commonly affecting the ethnic groups originating from around the Mediterranean Sea, including Armenians, North African Arabs, Sephardic Jews, and Turks. It is the most prevalent hereditary periodic fever worldwide characterized by self-limited recurrent episodes of fever (FMF attack) and is often complicated by amyloidosis. FMF is caused by mutations in *MEFV *gene coding for the protein pyrin. Although the genetic defect is known, the mechanisms by which this defect causes inflammatory attacks are largely unclear [1,2].

Pyrin contains a 92-aminoacid N-terminal pyrin domain that is shared by a number of other proteins involved in apoptotic and inflammatory signaling pathways. The pyrin domain is a member of the six-helix bundle, death-domain superfamily that includes death domains, death effector domains, and caspase recruitment domains (CARDs) involved in the protein-protein interactions required for apoptosis or signaling through NFκB. Therefore, it is proposed that pyrin is likely a part of regulatory pathway of inflammation and normally assists in keeping inflammation under control by deactivating the immune response and uncontrolled inflammation. Apoptosis plays an important role in down-regulation of the inflammatory response by reducing the lifespan of activated immunocompetent cells. Thus, increased apoptosis may be associated with pathogenesis of FMF [3-6].

Flow cytometry study indicated that neutrophil and monocyte apoptosis is significantly increased in FMF patients as compared to control subjects [7]. Since no difference in lymphocyte apoptosis between FMF patients and norm was found [8], and animal studies demonstrated decreased macrophage apoptosis in FMF [9], this finding may indicate that FMF is an autoinflammation of certain peripheral cells. On the other hand, this finding may also reflect defect in apoptosis regulation in FMF-patients. Further studies are required to understand the significance of apoptosis in the process of FMF-related inflammation.

Since 1972 colchicine treatment is the only prophylaxis against FMF attacks, related inflammatory episodes and amyloidosis [1,2]. Colchicine prevents the activation of neutrophils and development of amyloidosis through inhibiting the assembly of microtubules and mitotic spindle formation by binding β-tubulin [1,2]. However, the exact way in which colchicine suppresses attacks is unclear. Our recent study demonstrated that regular colchicine treatment result in suppression of the complement system, the major mediator of the inflammatory immune response [10]. In the present study we determined the serum levels of apoptotic marker, Annexin A5 (ANX-V), in FMF-patients within an attack and attack-free in comparison to healthy volunteers group and assessed the influence of colchicine treatment on this parameter. In addition, in all study subjects serum levels of C-reactive protein (CRP) and interleukine-1β (IL-1β), and the total leukocyte count (TLC) were also determined; and. correlation between all measured parameters was analyzed.

## Materials and methods

Forty four FMF-affected subjects (female/male: 19/25; mean age ± SD: 30.28 ± 10.57; within attack/attack free: 20/24) and 50 age- and sex-matched healthy volunteers (controls) without family history of FMF or other autoinflammatory diseases were enrolled in this study. All subjects were Armenians living in Armenia. Subjects with concurrent diseases or conditions interfering with the aim of this study, such as inflammatory, infectious, or autoimmune diseases, vasculitis, myocardial infarction, cancer, hematological diseases, severe renal or liver failure, gynecologic or urologic diseases, any surgical interventions within the previous 12 months, and those on immune-modulator drugs, were not included in the study. The study was approved by the local Ethics Committee; all subjects gave informed consents to provide 5 ml of venous blood for this study. The patients were recruited from the Department of Internal Diseases (Hospital N1) of Yerevan State Medical University. Among them, 21 were colchicine-free (12 of them were colchicine-naïve, for the remainder of the patients colchicine-free interval was 2 years), 23 received colchicine regularly for at least 2 last years prior to this study. Ten patients had renal amyloidosis. The clinical diagnosis of FMF was based on the Tel-Hashomer criteria. [11]. All patients showed relevant to FMF pathogenic mutations of pyrin gene, including M694V, V726A, M680I (G/A), M680I (G/C), F479L, and E148Q. Most patients had more than one of these mutations, and were heterozygous for each mutation. Seven patients were homozygous for M694V. Practically fasting blood samples were collected by venipuncture at 9:00-10:00 a.m. in two aliquotes. One was immediately used for measuring TLC, another was kept on ice for 60 min, then centrifuged at 3000 g for 15 min at 4°C to separate serum. The obtained serum samples were stored in aliquots at -30°C and thawed immediately prior to use. Serum levels of ANX-V and IL-1β were determined by ELISA using commercially available kits (Hölzel Diagnostika GmbH, Germany and Vektor-Best, Russia, respectively), according to manufacturers' instructions. Concentration of CRP in the serum was measured by turbidimetric method (Hmalyzer 2000, HUMAN, Germany). TLC was measured by automated hematology analyzer. For data analysis ordinal descriptive statistics and Student's unpaired two-tailed t-test with Welch's correction (upon comparing unequal variances) were applied using "Graphpad Prism" (GraphPad Software Inc., USA) software. P-values < 0.05 were considered to be significant.

## Results

**Figure 1:**
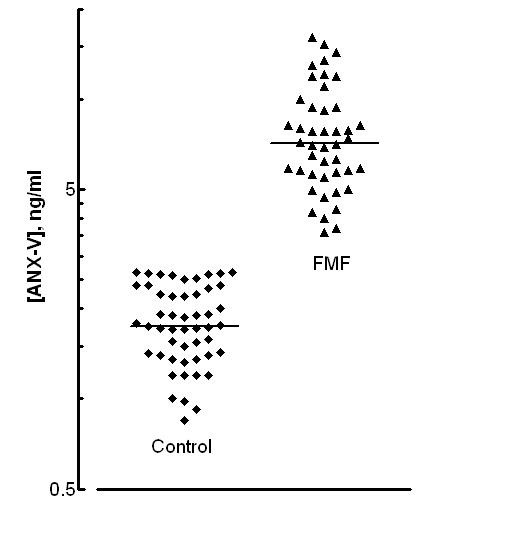
**Levels of ANX-V in patients with FMF and healthy controls**. Data are expressed in logarithmic scale as scatter plots, with the solid line representing the median value.

**Figure 2:**
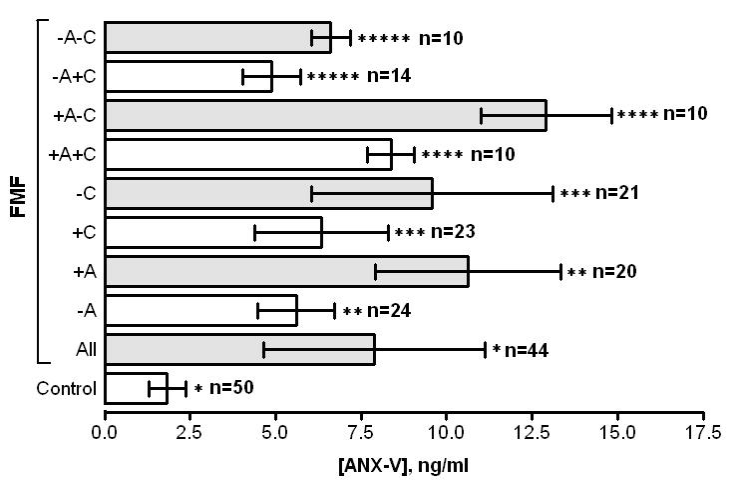
**Levels of ANX-V (M ± SD) in FMF patients depending on the stage of disease and treatment**. +A - patients within attack; -A - attack-free patients; +C - patients received regular colchicine treatment; -C - colchicine-free patients. * - p < 0.0001, ** - p < 0.0001, *** - p < 0.0009, **** - p < 0.0001, ***** - p < 0.0001.

According to the results obtained significantly increased serum levels of ANX-V were detected in all FMF patients as compared to controls (M ± SD: 7.88 ± 3.23 vs 1.83 ± 0.53, p < 0.0001), with higher levels in the attack group compared to attack free (M ± SD: 10.64 ± 2.72 vs 5.598 ± 1.11, p < 0.0001). Patients receiving regular colchicine treatment have significantly lower levels of ANX-V in comparison to those colchicine-free (6.341 ± 1.96 vs 9.567 ± 3.53, p < 0.0009). The results obtained are summarized on Figures 1, 2.

All patients have significantly higher CRP level (M ± SD: 59.17 ± 14.92 vs 1.5 ± 0.2, p < 0.0001), IL-1β level (M ± SD: 5.281 ± 3.2 vs 2 ± 1.2, p < 0.002), and TLC (M ± SD: 12800 ± 2621 vs 6400 ± 1032, p < 0.001) in comparison to the healthy subjects, with higher values in attack patients and lower in colchicine-free patients.

## Discussion

ANX-V, belonging to a recently discovered family of proteins, the annexins, has proven to be a useful tool in detecting apoptotic cells, since it preferentially binds to negatively charged phospholipids like phosphatidylserines (PS) in the presence of Ca2+. ANX-V is an important modulator of the immune response against PS-exposing particles like apoptotic cells, necrotic cells, and certain viruses. Loss of plasma membrane asymmetry, resulting in the exposure of PS, is considered to be an early event in apoptosis and is detected before morphological changes associated with apoptosis have occurred and before membrane integrity has been lost. The exposure of PS is one major "eat me" signal for phagocytes of apoptotic cells, which are normally cleared via an anti-inflammatory pathway. ANX-V may interfere in vivo with the immunosuppressive effects of apoptotic cells, since it preferentially binds PS with high affinity and inhibits apoptotic cell uptake by phagocytes [12,13].

ANX-V is produced by a variety of cells and is an in vivo marker of cellular injury and death. Circulating ANX-V can be released from the cells of the vascular wall (endothelial cells, smooth muscle cells), from secretor cells of the spleen and liver or from apoptotic particles derived from injured tissue, monocytes, lymphocytes, etc. High blood levels of ANX- V reflect high rate of cell death and the severity of cell damage [14,15].

The present study demonstrates that pathogenesis of FMF is associated with the increased levels of circulating ANX-V, especially in attack period, together with the increased levels of CRP and IL-1β, and TLC. Our data most probably reflect raised production of ANX-V as a response to clear the unwanted inflammatory cells and an increased rate of cell death in FMF. Higher levels of ANX-V in FMF patients within attack may be considered as an explanation of the self-limited nature of the FMF attacks [1,2]. As far as increased levels of circulating ANX-V were detected not only during FMF-attack but also in attack-free period, this finding may also reflect the chronic nature of disease-related inflammation, and inability of defective pyrin to keep inflammation under control [1,2]. The results obtained confirm previous observation based upon flow-cytometry data indicating increased rate of apoptosis of subpopulations of leukocytes involved in autoinflammation and recurrent episodes of fever in FMF [7].

In addition, our results demonstrated that regular colchicine treatment sufficiently decreases the levels of circulating ANX-V. This is probably due to suppressing effect of colchicine on leukocyte degranulation/chemotaxis and production of the inflammatory mediators, CRP and IL-1β, as revealed by this study, and on activity of the complement cascade, as it was shown in our previous report [10].

## Conclusions

Pathogenesis of FMF is characterized by the increased levels of circulating ANX-V, which is higher in patients within the attack and which reflect elevated rates of apoptosis of subpopulations of leukocytes involved in autoinflammation and recurrent episodes of fever, distinctive features of FMF. Regular colchicine treatment sufficiently decreases the rate of apoptosis in FMF patients by affecting the intensity of autoinflammatory reactions.

## Conflict of interest

The authors declare that they have no competing interests.

## Authors' contributions

AB generated the idea of the study, performed general supervision of the research works, and developed final version of the manuscript. GM participated in methodological design, performed statistical analysis, interpretation of data and drafting of manuscript. LH carried out collection of blood samples, preparation of serum samples, and performed immunoassays. TH performed CRP and TLC measurements.

All authors read and approved the final manuscript.
